# Is thymidylate synthase a reliable predictor for response and survival during hepatic arterial infusion for hepatic metastases from colorectal cancer?

**DOI:** 10.1038/sj.bjc.6603424

**Published:** 2006-10-17

**Authors:** G Ferretti, A Alimonti, F Cognetti

**Affiliations:** 1 Department of Medical Oncology, Division of Medical Oncology A, Regina Elena Cancer Institute Via Elio Chianesi 53, Rome 00144, Italy

**Sir**,

Decreased levels of the target enzyme thymidylate synthase (TS) have been repeatedly associated with superior clinical outcome in gastrointestinal cancers, including stomach cancer. In the study by Goekkurt *et al* (2006), patients who possessed a TS 5′ genotype associated with low TS mRNA expression levels showed a trend for superior survival time compared with those having TS genotypes associated with high TS mRNA expression. A significant association between clinical outcome to 5-fluorouracil (5FU)-based chemotherapy in colorectal cancer and TS polymorphisms was demonstrated when both TS polymorphisms within the 5′ untranslated (UTR) region were contemporarily analysed (Marcuello *et al*, 2004).

TS gene amplification and overexpression can lead to resistance to TS-targeting chemotherapeutic drugs (5FU and fluorodeoxyuridine (FUDR)) (Copur *et al*, 1995; Davies *et al*, 1999; Wu and Dolnick, 2003). Moreover, by hepatic arterial infusion (HAI) it is possible to reach lower TS mRNA and higher ribonucleotide reductase activity than with systemic chemotherapy (SC) (Kubota *et al*, 2002).

HAI therapy could generate higher intracellular levels of 5FU and FUDR metabolite, fluorodeoxyuridylate, locally (Gonen *et al*, 2003a). Among 135 patients randomly assigned to receive HAI *vs* systemic bolus 5FU and leucovorin, overall survival was significantly longer for HAI *vs* systemic treatment (*P*=0.003), as was time to hepatic progression (*P*=0.03) (Kemeny *et al*, 2006). By contrast, time to extrahepatic progression was significantly shorter in the HAI group (*P*=0.02).

In a previous study, Gonen *et al* (2003b) reported that patients with resectable TS overexpressing (TS+) liver metastases from colorectal cancer have better overall survival (OS) when treated by HAI plus SC rather than by SC alone. On the contrary, patients with TS-negative metastatic colorectal cancer treated with SC plus HAI had similar OS compared with SC alone. More interestingly, in the study by Kemeny *et al* (2006), liver biopsies in the HAI group, although based on small numbers (40 patients), demonstrated that for patients with TS levels in tumour ⩾4 the median OS was 14 months, while for those with levels less than 4, median OS was 24 months.

As previously reported (Alimonti *et al*, 2003), we think that, regarding patients treated with SC, it could be interesting to ascertain the role of the thymidine phosporylase (TP), the first enzyme involved in the metabolic activation pathway of FU to fluorodeoxyribonucleotides. Conversely, continuous HAI of FUDR generates FUDR monophosphate via uptake and activation by thymidine kinase and, unlike i.v bolus 5FU treatment, levels of TP would not likely be of predictive value. Preoperative biopsies and resection specimens from patients with stage II/III rectal carcinoma receiving neoadjuvant 5FU-based chemoradiotherapy have been recently studied for TS and TP protein expression by immunohistochemistry, and results have been compared with histopathologic tumour regression. A significant association was observed between high TS expression in tumour biopsies as well as resection specimens and nonresponse of the tumour to therapy (*P*=0.04), but low TP expression in the resection specimens was significantly associated with lack of response (*P*=0.02) (Jakob *et al*, 2005). However, it is still controversial whether TP expression in tumour is an independent factor that adds to TS positivity in worsening the prognosis of patients with metastatic colorectal cancer.

Interindividual variation in the activity of metabolising enzymes can affect the extent of pyrimidine prodrug activation, acting on the efficacy of chemotherapy treatment (Maring *et al*, 2005). Patients with low levels of TP could be unable to properly metabolise FU administered by i.v. systemic infusion. A repeat polymorphism within the 5′ UTR, that alters TS expression, has been correlated with response and survival in colorectal cancer patients receiving 5FU in several studies (Iacopetta *et al*, 2001). Thus, the role of germline polymorphisms (uridine monophosphate kinase (UMPK), orotate phosphoribosyl transferase (OPRT), TS, dihydropyrimidine dehydrogenase (DPD), and methylene tetrahydrofolate reductase (MTHFR)), tumour-specific somatic mutations and gene/protein expression levels (OPRT, UMPK, TS, DPD, uridine phosphorylase, uridine kinase, TP, thymidine kinase, deoxyuridine triphosphate nucleotide hydrolase) must be taken into account in explaining variation in anti-tumour efficacy and toxicity of 5F.
